# Oophorectomy in Cancer

**Published:** 1898-06-18

**Authors:** 


### OOPHORECTOMY IN CANCER.

Dr. Herman relates a case in which cancerous growths surrounding the axillary vessels, extending above the clavicle, and fixed to the subclavian vein, subsided and, indeed, went away after the performance of oöphorectomy and the administration of thyroid extract, the treatment suggested by Dr. Beatson. The case is especially interesting from the fact that, while some cases have recently been reported in which the operation of oöphorectomy alone has not been attended with any useful results, both this patient and the one operated on by Dr. Beatson took thyroid as well as having the operation performed. Dr. Herman's patient was a woman forty years of age, whose left breast had been removed for cancer. The whole of the mamma had been removed, but, as no glands were discovered in the axilla, the axillary space was not evacuated. This operation was done by Mr. Treves in October, 1890, and the patient recovered well. In the beginning of 1896, however, she began to lose flesh, but gained it again. Towards the end of December, 1896, however, she noticed a lump in the armpit, and on January 4th Mr. Treves cut down in the first instance upon a mass which lay just above the clavicle, and then upon that in the armpit. Both of these were found to be fixed to the vessels; there were also nodules in the pectoralis major, and the growth taken altogether was too extensive to permit complete removal; the incisions were therefore closed. The wounds healed well. Menstruation had been irregular for about three years, but had not ceased; the last period, in fact, had occurred on January 3rd, 1897. On March 2nd, 1897, both ovaries were removed, and then thyroid was commenced and has been continued ever since, the quantity varying from ten to fifteen grains a day. In September it was noted that the supra-clavicular nodule was no longer to be felt and that the axillary artery was apparently quite free from growth around it, and in May of this year it was reported that the scars were soft, that there was no adhesion to the subjacent parts, and that there were no deposits of any kind in the surrounding tissues. Such a condition of affairs fourteen months after the operation certainly seems to justify the assertion that the course of the disease has been modified by the treatment adopted.

